# Negative Alterations in the Respiratory Activity of Isolated Crude Heart Mitochondria Following In Vivo Isoproterenol Injection in Rats Are Not Observed in Heart Homogenate Suggesting That the Isolation Procedure Generates Experimental Artefacts

**DOI:** 10.3390/ijms26062388

**Published:** 2025-03-07

**Authors:** Dairo Alonso Rendon

**Affiliations:** Laboratorio de Biofísica, Departamento de Física, Facultad de Ciencias, Universidad Nacional de Colombia—Sede Medellín, Medellín AA3840, Colombia; darendon@unal.edu.co; Tel.: +57-4-430-93-78

**Keywords:** isoproterenol, heart mitochondria, oxidative phosphorylation, mitochondrial respiration, infarct, Takotsubo cardiomyopathy, method

## Abstract

Mitochondrial respiratory parameters (state 2 mitochondrial respiratory activity (state 2), state 3 mitochondrial respiratory activity (state 3), respiratory control (RC), mitochondrial ATP synthetic activity (MASA), and oxidative phosphorylation efficiency (ADP:O)) were assayed in heart homogenates (HHs) and in unwashed isolated mitochondria (isolated crude heart mitochondria (CHMs)), using rats sacrificed 3, 6, 24, and 48 h after receiving a subcutaneous injection of (−)-isoproterenol (67 mg/kg body weight). With HHs, the following was observed: (a) a statistically significant activation of RC and MASA at 3 h and 6 h after drug infusion; at those times, state 2, state 3, and ADP:O were not different. (b) No studied (−)-isoproterenol mitochondrial parameters were statistically different at 24 h and 48 h after drug administration. So extrapolating, (−)-isoproterenol treatment does not negatively impact mitochondrial respiratory function in vivo; on the contrary, a better 3 h and 6 h (−)-isoproterenol mitochondrial energetic functional state was observed. With CHMs, the following was observed: (a) a statistically significant activation of RC and MASA at 3 h, but no longer at 6 h after drug infusion. (b) No studied mitochondrial parameters were statistically different at 24 h after (−)-isoproterenol treatment, but at 48 h, a statistical decrease took place in (−)-isoproterenol RC, so the mitochondrial isolation procedure (MIP) causes additional negative alterations to the mitochondrial samples; therefore, isoproterenol-induced negative alterations of mitochondrial respiratory parameters reported in the literature using isolated heart mitochondria (IHMs) are possibly an experimental artefact.

## 1. Introduction

Isoproterenol is a synthetic β-adrenergic agonist, which interacts with the β-adrenergic receptor on the target cell’s external surface [1]. This agonist-receptor complex activates the adenyl cyclase enzyme on the internal surface of the plasma membrane, increasing the cytoplasmic cyclic AMP concentration, which then inhibits or stimulates many intracellular processes. There are two isoproterenol optical isomers: (−)-isoproterenol and (+)-isoproterenol. (±)-isoproterenol is a mixture (in equal proportions) of the two above-mentioned optical isomers. (−)-isoproterenol is the isomer activator of the β-adrenergic receptors.

The clinical use of catecholamine L-norepinephrine to maintain the blood pressure entails focal myocarditis and pericardium/endocardium hemorrhagic lesions [2]. Based on this clinical observation, Rona and co-workers carried out their outstanding research [3], in which an infarct-like myocardial lesion in rats was modeled using isoproterenol (a synthetic catecholamine). It was established that isoproterenol induces myocardial cell injury similar to that reported for myocardial infarction, myocardial ischemia, Chagas cardiomyopathy, and Takotsubo cardiomyopathy [4,5,6,7,8,9]. At present (based in PubMed literature) the (−)-isoproterenol treated rats constitute a widely used model of an infarct-like myocardial lesion.

Because mitochondrial bioenergetics is at the very center of heart metabolism, heart mitochondria have been the object of intensive studies of various myocardial pathologies. In isoproterenol heart research, isoproterenol-induced negative alterations of the heart mitochondrial bioenergetic parameters have been reported [7,10,11,12,13,14,15,16,17,18,19]. In particular, Curti et al. [15], using rat IHMs, report having observed negative alterations in the (±)-isoproterenol mitochondrial ATPase hydrolytic activity (MAHA). However, when HHs were used, the MAHA and MASA were not negatively modified by (±)-isoproterenol treatment [20,21]. Furthermore, an activation of the MASA occurred sometime after (±)-isoproterenol administration [21]. In this way, the results of the studies [20,21] disagree with the main conclusion of Curti et al. [15], who considered that negative alterations in the mitochondrial ATPase structure take place during (±)-isoproterenol-induced myocardium injury. Since IHMs were used in the research [15], in the investigations [20,21] it is concluded that the (±)-isoproterenol mitochondrial ATPase kinetic alterations observed by Curti et al. [15] themselves could have been additionally caused by the MIP. Later, Chagoya de Sanchez et al. [7] and Uyemura et al. [16] studied the respiratory activity of IHMs obtained from (−)-isoproterenol- and (±)-isoproterenol-injected rats. In these investigations [7,16], negative alterations of the heart mitochondrial respiratory activity (HMRA) upon isoproterenol treatment were reported. Uyemura et al. [16] reaffirmed (and complemented) their hypothesis, already put forth by them in their investigation [15] of negative structural alterations in vivo of (±)-isoproterenol heart mitochondrial ATPase (and in the (±)-isoproterenol heart inner mitochondrial membrane). The main objective of the present study is to show if the MIP itself causes negative changes in the structural-functional state of the heart mitochondria isolated from (−)-isoproterenol-treated rats. Therefore, the HMRA in the following biological samples was studied: (i) in the first (labelled HHs), resulting from the first step (the homogenization of the heart tissue) in the mitochondrial isolation, (ii) in the second (labelled CHMs), which is obtained by centrifuging the HHs, centrifuging the supernatant again, and finally collecting the resulting pellet; and (iii) in the third (labelled IHMs), obtained on re-suspending the CHMs, centrifuging this new suspension, and collecting the pellet.

## 2. Results

**Figure 1:**
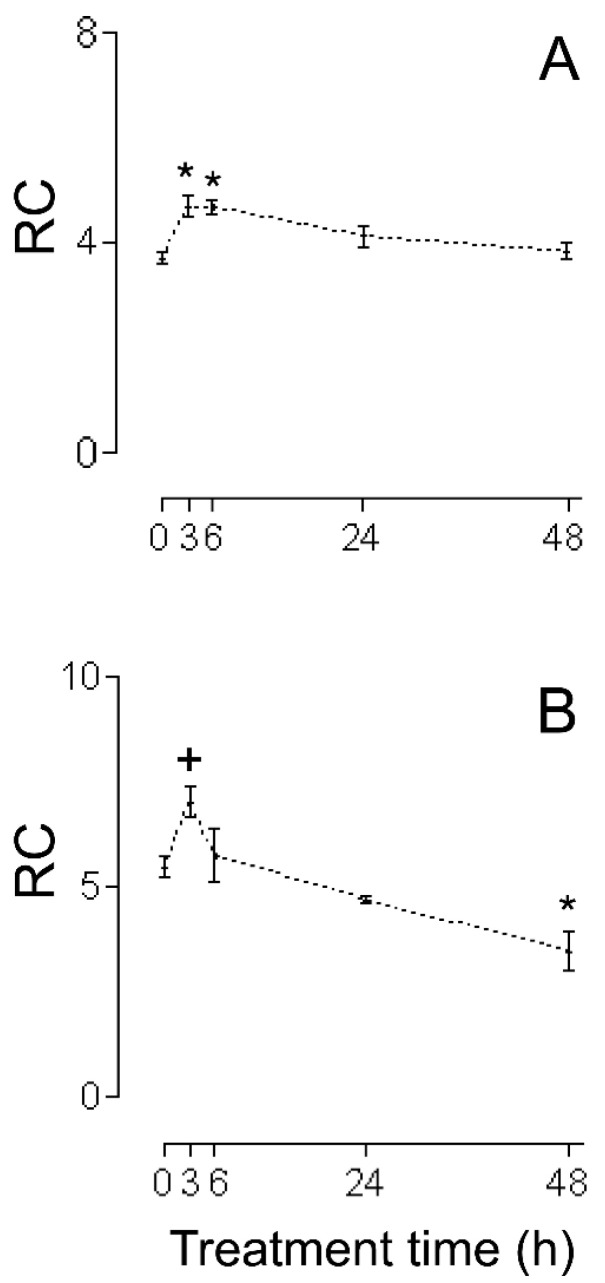
RC (dimensionless) during cellular lesion of myocardium by (−)-isoproterenol. (**A**) HHs, (**B**) CHMs. The control group is when the time after (−)-isoproterenol dose is 0 h. All data are averages ±SEM (n = 5 for HHs; for (−)-isoproterenol CHMs: control group n = 6; n = 5 for 3, and 6 h after treatment; n = 4 for 24, and 48 h after treatment). Significant statistical difference from the respective control group: * (*p* < 0.01) and + (*p* < 0.05).

**Figure 2:**
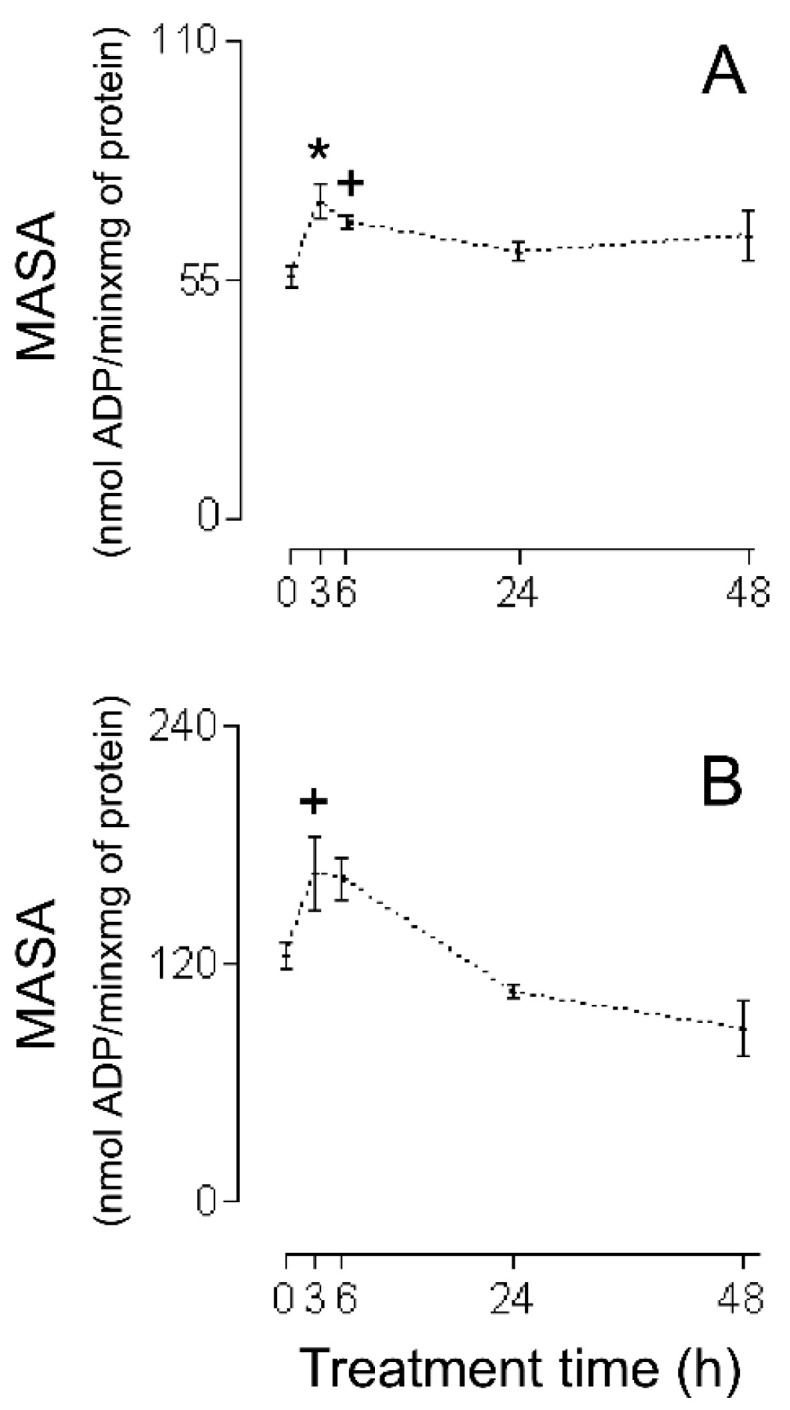
MASA during cellular lesion of myocardium by (−)-isoproterenol. (**A**) HHs, (**B**) CHMs. The control group is when the time after. (−)-isoproterenol dose is 0 h. All data are averages ±SEM (n = 5 for HHs; for (−)-isoproterenol CHMs: control group n = 6; n = 5 for 3, and 6 h after treatment; n = 4 for 24, and 48 h after treatment). Significant statistical difference from the respective control group: * (*p* < 0.01) and + (*p* < 0.05).

**Figure 3:**
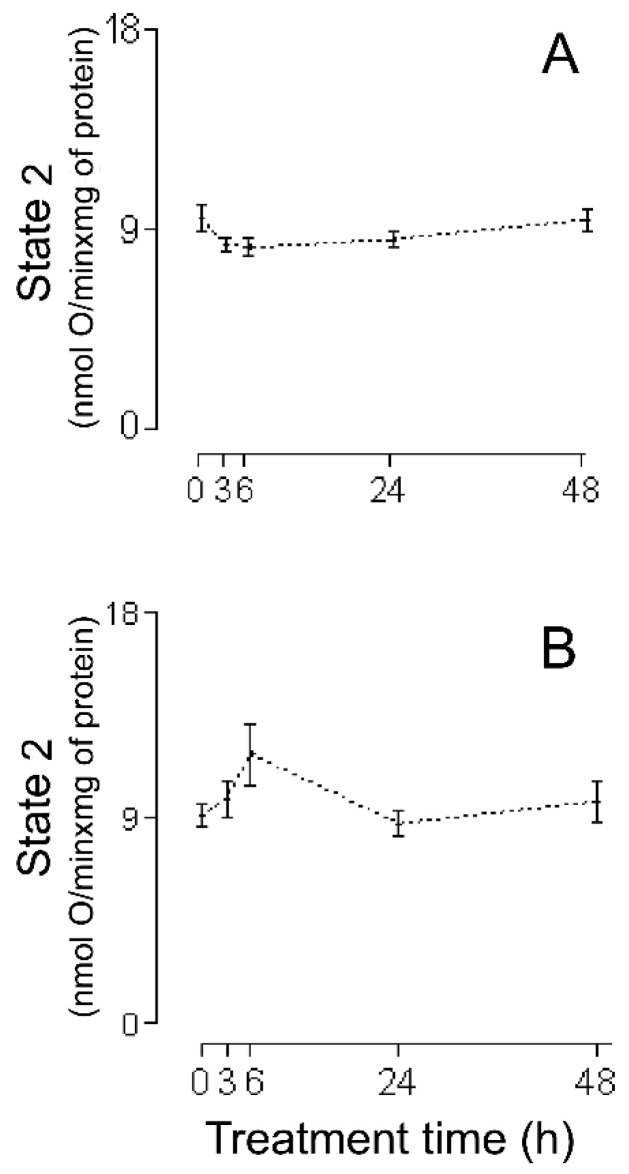
State 2 during cellular lesion of myocardium by (−)-isoproterenol. (**A**) HHs, (**B**) CHMs. The control group is when the time after (−)-isoproterenol dose is 0 h. All data are averages ± SEM (n = 5 for HHs; for (−)-isoproterenol CHMs: control group n = 6; n = 5 for 3, and 6 h after treatment; n = 4 for 24, and 48 h after treatment).

**Figure 4:**
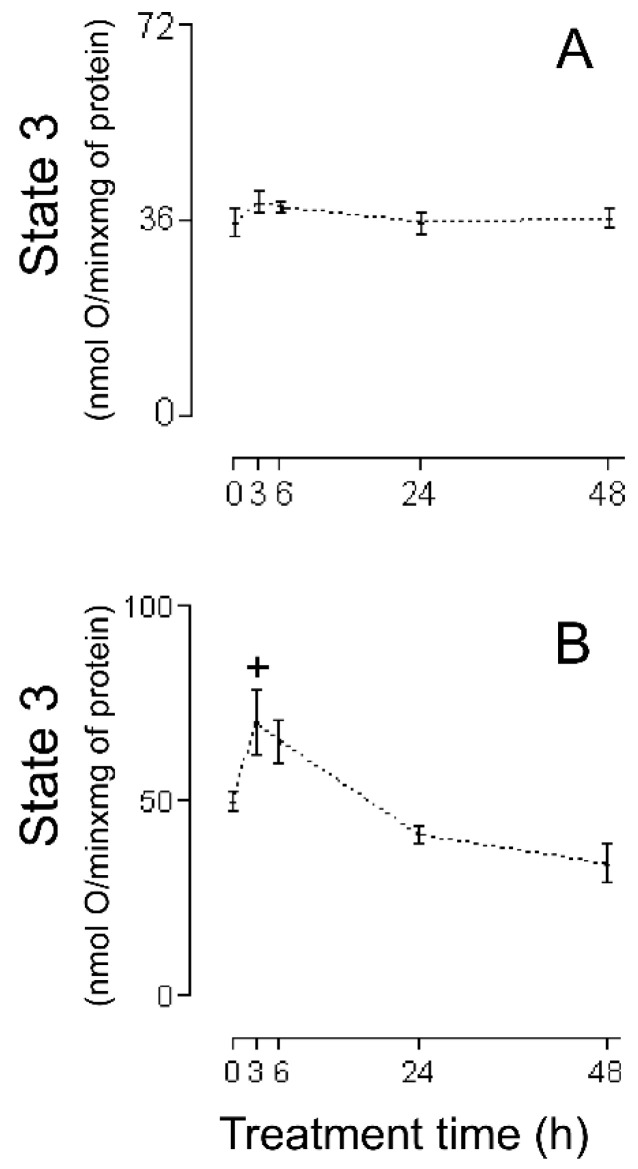
State 3 during cellular lesion of myocardium by (−)-isoproterenol. (**A**) HHs, (**B**) CHMs. The control group is when the time after (−)-isoproterenol dose is 0 h. All data are averages ±SEM (n = 5 for HHs; for (−)-isoproterenol CHMs: control group n = 6; n = 5 for 3, and 6 h after treatment; n = 4 for 24, and 48 h after treatment). Significant statistical difference from the respective control group: + (*p* < 0.05).

**Figure 5:**
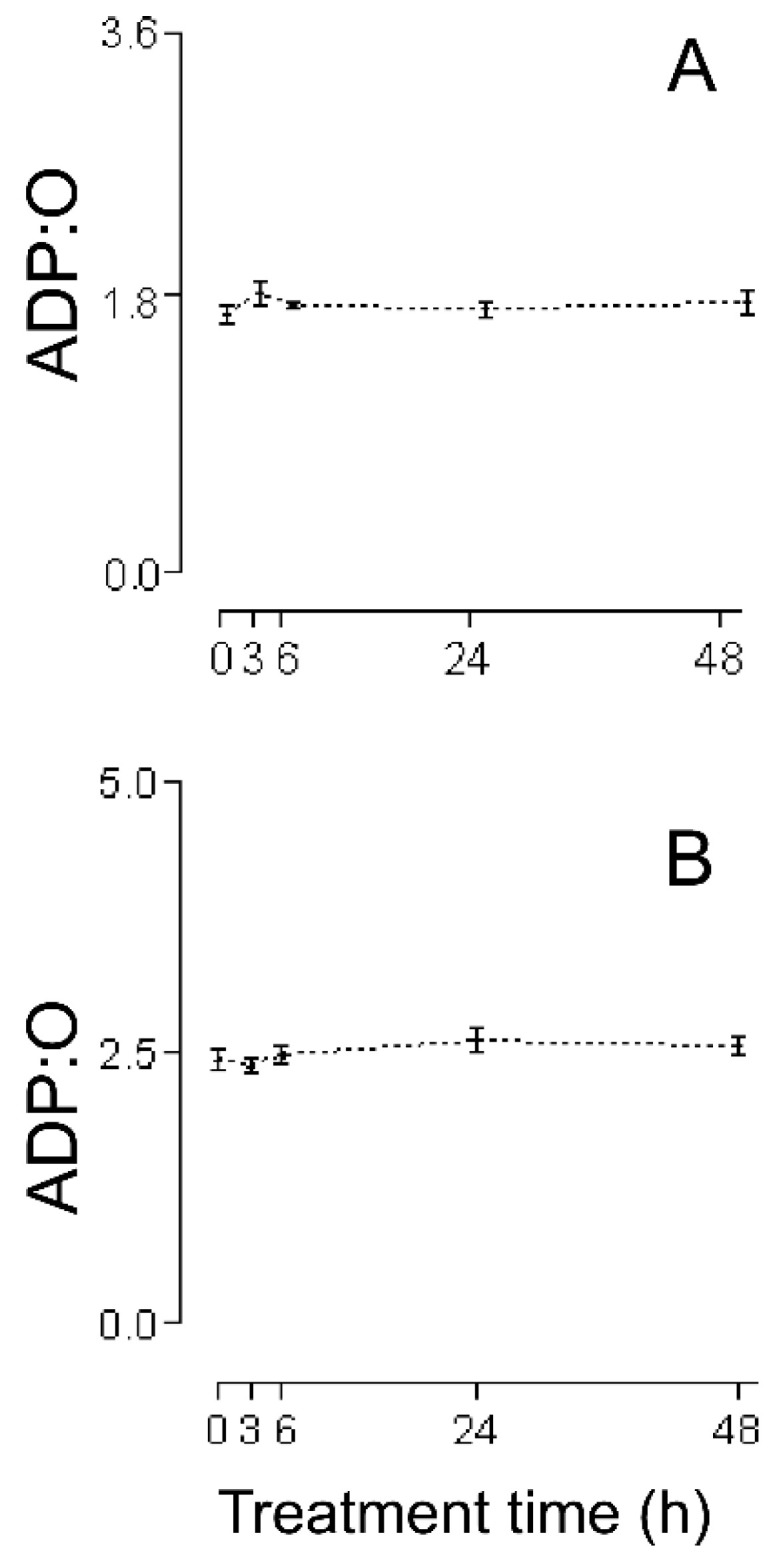
ADP:O (dimensionless) during cellular lesion of myocardium by (−)-isoproterenol. (**A**) HHs, (**B**) CHMs. The control group is when the time after (−)-isoproterenol dose is 0 h. All data are averages ±SEM (n = 5 for HHs; for (−)-isoproterenol CHMs: control group n = 6; n = 5 for 3, and 6 h after treatment; n = 4 for 24, and 48 h after treatment).

The first series of experiments consisted of an evaluation of the HMRA using HHs (Figure 1A (RC), Figure 2A (MASA), Figure 3A (state 2), Figure 4A (state 3), and Figure 5A (ADP:O)). In these figures, the averages of the values for each of the (−)-isoproterenol groups were never less than their respective controls. The increase in the RC for the groups in which the (−)-isoproterenol-treated rats were sacrificed at 3 or 6 h after the treatment (Figure 1A) should be emphasized, this being an indication of improved energetic functioning in vivo of the (−)-isoproterenol heart mitochondria (at the aforementioned times).

To evaluate the possible influence of the MIP in the energetic-functional state of the (−)-isoproterenol heart mitochondria, we proceeded to determine the HMRA in the mitochondrial samples obtained after the first stage of the MIP (the CHMs; Figure 1B (RC), Figure 2B (MASA), Figure 3B (state 2), Figure 4B (state3), and Figure 5B (ADP:O)). Then for this second series of experiments, only on the 3 h (−)-isoproterenol group was an increased RC maintained (Figure 1B), but what was most notable was the decrease in the RC at 48 h after the (−)-isoproterenol treatment (Figure 1B) (confirming a negative influence by the MIP on the energetic-functional state of these heart mitochondrial samples).

**Figure 6:**
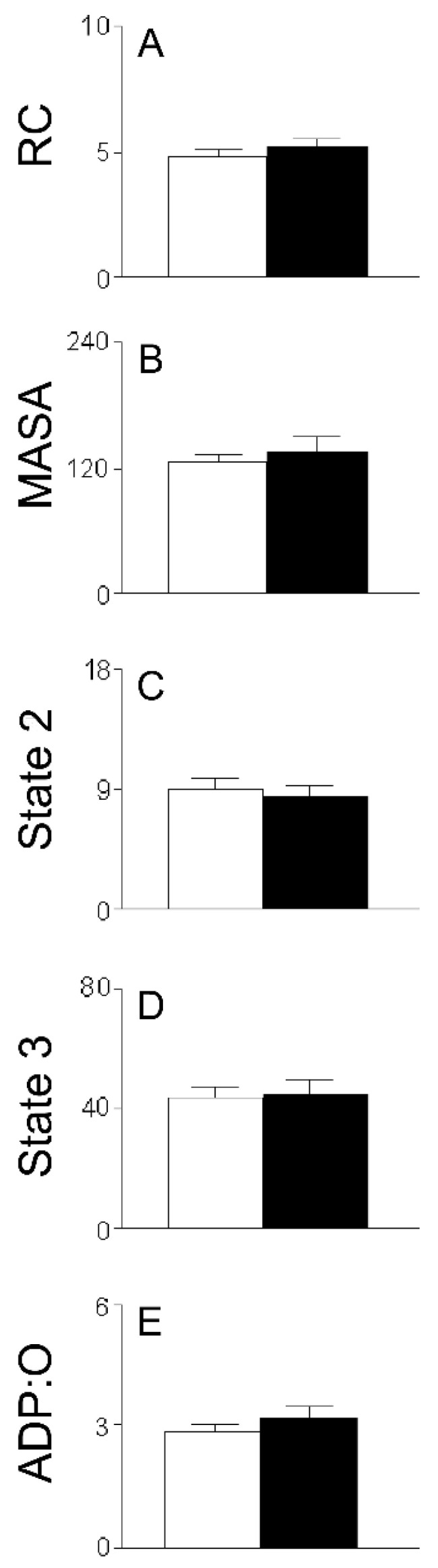
(**A**): RC (dimensionless), (**B**): MASA (nmol ADP/minxmg of protein), (**C**): state 2 (nmol O/minxmg of protein), (**D**): state 3 (nmol O/minxmg of protein), and (**E**): ADP:O (dimensionless) in IHMs at 3 h after (−)-isoproterenol treatment. All data are averages ± SEM (n = 6 for control group; n = 4 for 3 h (−)-isoproterenol isolated heart mitochondria).

Due to the fact that for the 3 h (−)-isoproterenol group, the first stage of the MIP did not negatively influence the RC (Figure 1A,B), it was pertinent to evaluate the HMRA in the 3 h (−)-isoproterenol heart mitochondria obtained after the second stage of the MIP (the IHMs; Figure 6). The data shows (Figure 1B and Figure 6) a negative influence of this MIP step on the energetic-functional state of the 3 h (−)-isoproterenol heart mitochondria.

## 3. Discussion

### 3.1. HMRA Using HHs

The data in Figure 1A show that the RC of all of the (−)-isoproterenol HH groups is never statistically lower than the control RC; furthermore, these data display a statistical increase in (−)-isoproterenol RC at 3 h and 6 h (26% in both cases) after drug administration. Due to the fact that the RC is the only mitochondrial respiratory parameter actually responsible for the mitochondrial energetic functional state (MEFS) [22], an extrapolation (to an in vivo situation) of the aforementioned results indicates that not only are no in vivo negative alterations in the MEFS of (−)-isoproterenol heart mitochondria exhibited, but that in vivo heart mitochondria have better MEFS at 3 h and 6 h after (−)-isoproterenol administration.

This improved MEFS is reflected in a statistical increase in the HH MASA at 3 h and 6 h (31% and 22%, respectively) after (−)-isoproterenol treatment (Figure 2A; the results in Figure 2A also show that in each of the other HH groups, the (−)-isoproterenol MASA is never statistically different from the control MASA).

An adequate analysis of the behavior of the mitochondrial respiratory parameters of state 2 and state 3 during a particular animal treatment allows clarifying the mechanism by which mitochondria have improved or worsened their MEFS [23]. In the present investigation using HHs, a slight but not statistically significant decrease (of 12% and 14%, respectively) can be seen (Figure 3A) in homogenate state 2 at 3 h and 6 h after (−)-isoproterenol administration (in these same treatment times, (−)-isoproterenol homogenate state 3 increases slightly but not statistically significantly by 10% and 8%, respectively; see Figure 4A).

All of this leads to the conclusion that state 2, as well as state 3, is responsible for the statistical increase at 3 h and 6 h of (−)-isoproterenol RC using HHs (Figure 1A; of all of the existing possibilities [23], the one seen in the present study with HHs is the most curious, in which state 2 falls while state 3 rises). State 2 is associated with the H^+^ mitochondrial leak (ML) from the intermembrane space toward the interior of the mitochondrial matrix; the larger the ML, the greater state 2. Generally, the ML takes place through the inner mitochondrial membrane lipid bilayer and/or the mitochondrial ATPase. Since state 2’s tendency to decrease is in part responsible for the better MEFS in HHs at 3 h and 6 h after (−)-isoproterenol administration, this tendency to decrease is achieved at the expense of a decrease in the ML (a curious action on the part of the (−)-isoproterenol treatment). In order to try to find the possible basic entity of the relation between a (−)-isoproterenol treatment and the ML, it is important to mention the following experimental observations: (i) An isoproterenol treatment causes an excessive energy demand as a result of a large increase in cardiac work [24] and the myocardium has the ability to balance the rate of energy conversion (i.e., ATP production by the mitochondrial ATPase) and heart work (i.e., controlled ATP hydrolysis by the myosin adenosinetriphosphatase) along a wide range of heart workloads [25]. (ii) Cardiomyocytes and H9c2 cells (a cell line from rat heart tissue) experience an increase in MASA because of the presence of isoproterenol [26,27], which is associated [26] with an increase in the intramitochondrial calcium concentration (IMCC). (iii) A physiological increase in the IMCC directly activates the heart mitochondrial ATPsynthase [28]. (iv) The intramitochondrial calcium causes structural changes in the mitochondrial ATPsynthase [29]. (v) Díaz-Muñoz et al. [30], using the same (−)-isoproterenol treatment as that employed in [7] (and so in the current study), showed a statistical increase in the IMCC at 3 h and 6 h after drug administration. On the basis of all of these experimental findings, in the present study it is considered that the following takes place: (−)-isoproterenol caused a large increase in cardiac work at 3 h and 6 h after drug administration, involving a statistical increase at these times in the HH RC and HH MASA (Figure 1A and Figure 2A); and (ii) the above was mediated by a statistical increase in the IMCC. Now, what has been stated occurred due to calcium-induced structural changes in the heart mitochondrial ATPsynthase [29], possibly involving a decrease in the ML through this mitochondrial complex in the absence of ADP (and thus the slight decrease, although not statistically significant, in the 3 h and 6 h (−)-isoproterenol homogenate state 2 (Figure 3A)). If the above-mentioned ML decrease had also taken place in the presence of ADP, then a decrease in the 3 h and 6 h (−)-isoproterenol homogenate state 3 should have been observed, but the results in Figure 4A show the contrary. In the presence of ADP, an absence of ML has been reported [31]. Thus, it is possibly as if another explanation for the slight increase, although not statistically significant, in the 3 h and 6 h (−)-isoproterenol homogenate state 3 (Figure 4A) is the physiological increase in the IMCC [28], fruit of which structural changes in the heart mitochondrial ATPsynthase take place [28]. With the foregoing, we also ended up finding an explanation for the aforementioned curiosity: because of the action of the (−)-isoproterenol treatment, the (−)-isoproterenol homogenate state 2 falls while the (−)-isoproterenol homogenate state 3 rises. At 24 h and 48 h after (−)-isoproterenol infusion, RC and MASA are not statistically different, in HHs, from their respective controls (Figure 1A and Figure 2A), suggesting that at those times heart work tends to reach normal values. Finally, the data of Figure 5A showed no statistical differences between the ADP:O of control HHs and that of (−)-isoproterenol HHs during the whole long-term study, confirming the fact [22] that the ADP:O parameter is not as sensitive as is the RC for detecting mitochondrial energetic functional changes.

In conclusion, studies with HHs show no negative impact on heart MEFS caused in vivo by (−)-isoproterenol treatment. On the contrary, at certain times after the drug is administered, an improved heart MEFS has been observed. In this context, it is very important to again mention the studies by Das and Harris [26] and by De Rasmo D et al. [27], in which an increase in the MASA is reported when the cardiomyocytes and H9c2 cells are exposed to isoproterenol, respectively. Here it also worthwhile referencing the research by Nef et al. [32], in which mitochondrial energy metabolism genes showed upregulation in patients with Takotsubo cardiomyopathy (characterized by very high plasma catecholamine concentration; the isoproterenol is a synthetic catecholamine); Nef et al. [32] considered the aforementioned upregulation to be crucial for the normalization of the contractile function of the heart achieved after this cardiomyopathy.

### 3.2. HMRA Using CHMs

The lack of deterioration of the MEFS of the (−)-isoproterenol homogenate mitochondria does not concord with the negative heart energetic mitochondrial alterations reported using IHMs prepared from isoproterenol-treated animals [7,10,11,12,13,14,15,16,17,18,19]. Because of this, it has been suggested that possibly the method of carrying out mitochondrial isolation was causing additional negative changes to the biological samples. In order to test this hypothesis, studies were carried out using CHMs. The RC data obtained using CHMs (Figure 1B) showed the following: (a) the RC increased statistically at 3 h after (−)-isoproterenol injection (as was seen in the studies with HHs, Figure 1A); (b) the RC was unchanged at 6 h after drug treatment (but at this treatment time in HHs the RC increased statistically, Figure 1A); (c) the 24 h (−)-isoproterenol RC and the control RC were statistically the same (in agreement with the HH studies, Figure 1A); (d) the RC was statistically lower (36%) in the 48 h (−)-isoproterenol group than in the control group (in HHs, 48 h (−)-isoproterenol RC was statistically equal to the control, Figure 1A). Taken together, all these RC results (from HHs and CHMs, Figure 1A,B) reveal that the procedure for obtaining the CHMs causes (in two of the four (−)-isoproterenol groups under study) additional negative alterations in the heart MEFS. In the 48 h (−)-isoproterenol CHMs (those that were most impacted by the MIP), state 2 did undergo slight increase (non-statistically by 7%, Figure 3B), but state 3 fell considerably (but non-statistically by 32%, Figure 4B); thus, the energetic uncoupling of these CHMs was not due to increases in the ML, but rather to problems in the mitochondrial components directly related to the process of oxidative phosphorylation (the 48 h (−)-isoproterenol MASA also fell considerably (but non-statistically by 30%, Figure 2B)). The reported finding of oxidative stress toxins in blood from isoproterenol-treated rats [33] possibly also implies that the greater the (−)-isoproterenol treatment time, the greater the accumulation of these toxins. On the other hand, the last step in the preparation of the CHMs was carried out in the present study in a WB without any kind of calcium chelator (as was carried out by Chagoya de Sanchez and co-workers [7]), so during the process of isolation, a subgroup of the CHMs could have been capturing (with the help of endogenous substrates) extra calcium (non-physiological) from the medium. The highly excessive accumulation of oxidative stress toxins in blood that could have taken place at 48 h after drug treatment (and in association with the above-mentioned non-physiological extra intramitochondrial calcium) could have led to the greater negative impact, through the action of the MIP, on a subgroup of the 48 h (−)-isoproterenol CHMs (possibly involving the activation of the mitochondrial permeability transition (mPT); in this vein, it is worthwhile mentioning the research by Bernardi et al. [29], in which it is argued that intramitochondrial calcium, on directly interacting with mitochondrial ATPsynthase, and in association with other factors (including reactive oxygen species), determines the transition of this mitochondrial complex from an energy-conserving into an energy-dissipating device (because of the activation of the mPT)). Another option is the following: possibly the 48 h (−)-isoproterenol-treated CHM subgroup described above did not reach the point of undergoing mPT during its isolation, but remained very preconditioned to undergo this process. Now, during the determination of the HMRA this subgroup of 48 h (−)-isoproterenol-treated CHMs ended up undergoing mPT, because in the incubation buffer there is inorganic phosphate, which in association with an elevated intramitochondrial calcium concentration induces the mPT. In order to test this point of view, in future research it would be advisable to isolate the isoproterenol heart mitochondria using the antioxidant BHT (2–10 µM), and without fail use a calcium chelator (the most appropriate is EGTA [23]) in all of the buffers. Here it is worth mentioning the inappropriate use in the study [7], and therefore in the present one, of very hypertonic HB and WB. In this way, the mitochondria lose a great deal of water during their isolation (ending up with their interior being very hypertonic (hypertonic mitochondria)). Additionally, in the study [7] the RB used was hypotonic, so the hypertonic mitochondria undergo a significant flow of water from the exterior medium during the respiratory activity assay. Therefore, without fail, all of the buffers should be isotonic.

In the CHM data at 3 h (−)-isoproterenol treatment, the (−)-isoproterenol state 2 is statistically equal to the control (Figure 3B), but the (−)-isoproterenol state 3 is statistically greater (Figure 4B), all of this involving a statistical increase in the (−)-isoproterenol RC (Figure 1B), or in other words an improved MEFS of 3 h (−)-isoproterenol CHMs (this in accordance with what has already been postulated: an increase in the 3 h (−)-isoproterenol heart work load also involves a physiological increase in the IMCC, in order to increase the necessary production of the mitochondrial ATP (which is observed with the statistical increase in the 3 h (−)-isoproterenol MASA (Figure 2B). In the 6 h (−)-isoproterenol CHMs, both state 2 and state 3 increase, but not statistically (Figure 3B and Figure 4B); nevertheless, these increases are considerable (29% and 31%, respectively). The 6 h (−)-isoproterenol RC did not undergo any type of statistical variation, Figure 1B (that is to say, the 6 h (−)-isoproterenol treatment did not entail any type of alteration in the MEFS). Now, the 6 h (−)-isoproterenol state 2 is greater than the 3 h (−)-isoproterenol state 2 (by a non-statistical 20%, Figure 3B), while the 6 h (−)-isoproterenol state 3 is slightly lower than the 3 h (−)-isoproterenol state 3 (by a non-statistical 7%, Figure 4B), all of this leading to the 6 h (−)-isoproterenol RC’s being lower than the 3 h (−)-isoproterenol RC (by a non-statistical 18%, Figure 1B), or in other words, the 6 h (−)-isoproterenol CHMs tend to be more energetically uncoupled than the 3 h ones (and this energetic uncoupling tendency is basically related to the increase in the ML of the 6 h (−)-isoproterenol CHMs with respect to the 3 h ones). The 6 h (−)-isoproterenol MASA is non-statistically 31% greater than the control (Figure 2B), but the 6 h (−)-isoproterenol RC is non-statistically 5% greater than the control (Figure 1B). The aforementioned percentages should be more or less of the same magnitude. In order to explain the foregoing contradiction, the existence of two mitochondrial subgroups at 6 h is proposed: one with better (−)-isoproterenol MEFS, and the other totally energetically uncoupled (the “weakest” ending up negatively altered by the MIP, and for which the consumption of oxygen in state 3 is the same as in state 2). In this way, the 6 h state 3 (Figure 4B) would be the consumption of oxygen on the part of the two aforementioned mitochondrial subgroups and thus the 6 h (−)-isoproterenol state 3 would not be exclusively associated with the synthesis of ATP. Due to the fact that the MASA was determined indirectly (MASA = state 3 × ADP:O), what was stated for the 6 h (−)-isoproterenol state 3 and the slight non-statistical increase in the 6 h (−)-isoproterenol ADP:O (Figure 5B) contribute to obtaining the high non-statistical increase in the 6 h (−)-isoproterenol MASA (but this is an apparent increase in the mitochondrial ATP synthesis). In the same vein, the value of the 6 h RC (Figure 1B) would be reflecting a kind of “average” of the MEFS of the two aforementioned mitochondrial subgroups (the 6 h RC’s being slightly higher by 5%, although not statistically, with respect to the control, would be indicating that the contribution of the MEFS of the energetically uncoupled mitochondrial subgroup to the aforementioned “average” is less than that of the other subgroup, but significant). All of the foregoing meant that in the 3 h, but not in the 6 h (−)-isoproterenol CHMs, a statistical increase in the RC was observed, as occurred with the 3 h (−)-isoproterenol homogenate mitochondria (Figure 1A,B). This again accords with what has been postulated: the greater increase in the heart workload at 6 h (−)-isoproterenol treatment, with respect to that at 3 h, leads to the 6 h (−)-isoproterenol CHMs containing, with respect to the 3 h ones, a greater physiological IMCC (in agreement with study [28]). But during the mitochondrial isolation, both the 3 h and the 6 h (−)-isoproterenol heart mitochondria could have captured (with the help of endogenous substrates) extra calcium from the WB prepared without any calcium chelator, possibly leading to a greater energetic uncoupling of the 6 h than of the 3 h (−)-isoproterenol CHMs (because the former had more physiological IMCC in advance than the latter). In order to test the foregoing point of view, in future research it would be a good idea to isolate the (−)-isoproterenol CHMs, utilizing 1–2 µM Rut 360 in all of the buffers (and without fail EGTA, and because of what has already been stated also 2–10 µM BHT. The Rut 360 impedes the entrance of extra calcium from the exterior medium into the mitochondrial matrix, through the electrophoretic calcium uniporter). Figure 5B data revealed no statistical differences between the ADP:O in control CHMs and that in (−)-isoproterenol CHMs during the whole long-term study, indicating that the ADP:O parameter is not sensitive enough to detect the energetic functional changes, able to be seen with the help of the RC (Figure 1B). Finally, in the CHMs, the peculiarity observed in the HHs was not seen, in which a tendency of the (−)-isoproterenol homogenate state 2 to fall is presented (Figure 3A) while at the same time the (−)-isoproterenol homogenate state 3 tends to rise (Figure 4A). This shows the great importance of employing tissue homogenates (along with isolated mitochondria), in bioenergetic mitochondrial investigations aimed at elucidating the effect in vivo of the substance under study (the finding of investigation [34] is another example of the importance of the use of tissue homogenates).

In conclusion, on comparing the results of studies using HHs with those obtained employing CHMs, it can clearly be seen that the MIP involves additional negative changes to the (−)-isoproterenol MEFS. In order to obtain adequate (−)-isoproterenol heart isolated mitochondria (that reflect a behavior closer to reality in vivo), the use of EGTA, BHT, and Rut 360 in all of the mitochondrial isolation buffers is very important (and all of the mitochondrial buffers should without fail be isotonic). For this same end, all of the strategies proposed in the study [23] are also recommended. The manual press used in the present study is emphatically recommended (the detailed blueprint for its manufacture can be found in the supplementary material of the study [23]), with which the heart was sliced very rapidly (and in very small parts) (in order to then proceed with the preparation of the HHs and the CHMs). The usual way is to slice the heart with the help of scissors, but that way takes a lot of time, which can be counterproductive when handling tissues obtained from animals previously treated with an oxidative stress-induced drug under study (see a concrete example in the study [23]).

### 3.3. HMRA Using IHMs

With the aim of establishing if the stage following the obtaining of the CHMs (unwashed isolated mitochondria) also causes additional changes to the mitochondrial sample, we proceeded to carry out experiments with IHMs (once-washed isolated mitochondria). Only the control and the 3 h (−)-isoproterenol group were studied in IHMs (Figure 6). The data in Figure 6A (and in Figure 6B) revealed no statistical differences between the RC (and the MASA) of the control IHMs and that of the 3 h (−)-isoproterenol IHMs, or in other words, because of the action of the single-wash mitochondrial procedure, the improved MEFS that the 3 h (−)-isoproterenol CHMs enjoyed (Figure 1B) has been lost. Since the statistical activation in state 3 of 3 h (−)-isoproterenol CHMs (Figure 4B) no longer takes place in the 3 h (−)-isoproterenol IHMs (Figure 6D), and since the 3 h (−)-isoproterenol IHMs state 2 remained, as in the case of the 3 h (−)-isoproterenol CHMs, statistically equal to the control (Figure 3B and Figure 6C), the first mitochondrial washing negatively and directly influenced the components related to the process of oxidative phosphorylation (not the ML). So all of these RC data (Figure 1B and Figure 6A) indicate that an additional negative influence of the MIP on the (−)-isoproterenol MEFS depends on how many times the heart mitochondria have been washed. Along this vein, it is important to note that Chagoya de Sanchez and co-workers [7] reported a statistical RC decrease in the 6, 24, and 48 h (−)-isoproterenol groups, and in the 3 h (−)-isoproterenol group, the RC was statistically equal to the control; that is to say, in the study [7] negative RC alterations were reported in more (−)-isoproterenol studied groups than found in the current study CHMs. This can be explained by the fact that Chagoya de Sanchez and co-workers [7] washed the isolated heart mitochondria twice, which led to many more negative mitochondrial alterations.

Finally, here it is worthwhile to cite the investigations [35,36] in which the HMRA has been studied using saponin-skinned heart fibers (HFs) obtained from isoproterenol-treated rats. It is to be expected that the preparation of the HFs involves less negative impact on the isoproterenol mitochondria than that of the MIP [37]. The researchers [35,36] do not report the RC, but this parameter was calculated from Figure 2 of study [35] and the available data from the link at the bottom of the paper [36] (this was only possible for the glutamate plus malate substrates in [35] and the malate plus octanoyl carnitine in [36], because for the rest of the substrates used, the studies [35,36] do not report state 2 or state 4). Thus, in study [35]: control RC = 5.2 and isoproterenol RC = 5.9 (an increase of 13.4%; it was not possible to calculate the *p* value, because in Figure 2 of the study [35], only the average values are available), and in study [36]: control RC = 2.65 ± 0.50 and isoproterenol RC = 2.34 ± 0.26). These data show that in these studies [35,36], the in vivo action of the isoproterenol did not negatively impact the energetic functional state of the isoproterenol HFs mitochondria (IHFMs). In these same studies [35,36], the isoproterenol state 2, as well as the isoproterenol state 3, are significantly lower than their respective controls, and since the isoproterenol RC are not lower, this is a reflection [23] of a negative impact, possibly caused by the procedure for obtaining the HFs, in only one population of the IHFMs. The researchers [35] report a greater sensitivity of the IHFMs to the exogenous calcium, which is due (as was already explained) to the higher IMCC in these organelles (making them more prone to undergo negative changes during their manipulation).

### 3.4. Disadvantage in the Present Study

In the present research, female Wistar rats were used, but in the study by Chagoya de Sanchez and co-workers [7], male Wistar rats were used. Notwithstanding the above, the aforementioned negative alterations, observed in mitochondria isolated from isoproterenol-treated rats, took place during their isolation. For this reason, it is obvious to expect that what happened is independent of the sex and the species of laboratory animal used.

### 3.5. Other Considerations

All isoproterenol heart mitochondrial studies [7,10,11,12,13,14,15,16,17,18,19] have reported negative changes in the MEFS of isoproterenol heart isolated mitochondria. It is worthwhile to emphasize that the types of these negative changes reported in the investigations [7,10,11,12,13,14,15,16,17,18,19] are very disparate. Thus, even four decades ago, Grieve and co-workers [13] were very concerned about the discrepancies in the isoproterenol mitochondrial respiratory parameters obtained by different authors at that time. The origin of these discrepancies can be found in the following: it is possible that the experimental artefact that was found in the current study was also present in all reported isoproterenol heart isolated mitochondrial studies [7,10,11,12,13,14,15,16,17,18,19], but the features of this artefact are very diverse, because different mitochondrial isolation procedures have been used (along with different isoproterenol treatments), which have additionally and negatively altered the isoproterenol heart isolated mitochondria in different ways. Here, it is worth mentioning the study by Tamura K et al. [38], in which the ATP yield (measured directly, by the luciferin-luciferase method, in heart isolated mitochondria) did not vary during one isoproterenol treatment. Many studies report a beneficial action of various substances [35,39,40,41,42,43,44,45,46,47,48,49,50] (and of some procedures, such as calorie restriction [19] and pancreatectomy [36] (to induce diabetes)) for partially or totally recovering the normal MEFS of heart mitochondria (or HFs) isolated from isoproterenol-treated animals. With respect to all of these studies, the following should be noted: (i) Extrapolating the current RC results with HHs (Figure 1A, Figure 2A, Figure 3A, Figure 4A and Figure 5A), an (−)-isoproterenol treatment does not negatively alter the MEFS of heart mitochondria in vivo; therefore, a follow-up of the heart MEFS is not a criterion for studying the beneficial action of a substance (or of some procedure) against isoproterenol-induced myocardium lesions. (ii) What possibly occurs is a significant decrease (due to the action of the substance under study) of the concentration of oxidative stress toxins in the blood of isoproterenol-treated animals. Therefore, during the isolation of the heart mitochondria from isoproterenol-treated animals (which previously had been supplied with the substance under study, group A), these cellular organelles entered into contact with lower blood oxidative stress toxin concentration than those isolated from isoproterenol-treated animals (and those with no other previous treatment, group B). This is the reason that the isolated heart mitochondria of group A exhibit a better MEFS than those of group B. In particular, it is worth pointing out that the researchers [39,40,41] wash the isolated heart mitochondria and determine the HMRA in buffers without any calcium chelator (furthermore, the WB is slightly hypertonic and the RB is slightly hypotonic). (iii) Calorie restriction protects heart mitochondria from oxidative damage and lowers mitochondrial oxidative stress [19,51,52,53]. In this way, calorie-restricted heart mitochondria are preconditioned starting in vivo to offer greater resistance against undergoing structural-functional changes during their isolation in which there is the presence in vivo and/or in vitro of elements that cause oxidative stress. It is for this reason that in study [19], the decrease in the isoproterenol RC no longer occurs in mitochondria isolated from isoproterenol-treated rats previously subjected to calorie restriction. Here it is important to mention the following: on the basis of the data kindly provided by researcher Heberty di Tarso Fernandes Facundo from study [19], it was found that with respect to the control RC (3.34 ± 0.39; n = 5), the calorie-restricted CR (3.49 ± 0.85; n = 3) is greater by 4.4% and the calorie-restricted isoproterenol-treated CR (4.40 ± 1.18; n = 3) is greater by 31.7%. This 31.7% is not statistically different. Possibly in study [19] this could have been achieved if all of the groups under study had had n = 5; in that way there would have been the opportunity (in [19]) for evidence of a better energetic functional state of the calorie-restricted isoproterenol mitochondria (fruit of the isoproterenol treatment and not of the calorie restriction itself, for which the increase was 4.4%). A pancreatectomy desensitizes the β-adrenergic receptors (see references in [36]). When the isoproterenol was injected into pancreatectomized rats (PRs), state 2 and state 3 (in the IHFMs obtained from these isoproterenol-injected PRs) were now equal to those evaluated in the IHFMs obtained from the PRs [36]. This is to be expected: the PR β-adrenergic receptors are desensitized, so in the PRs the isoproterenol did not increase the IMCCs (which would have made them more prone to undergo artefactual changes, and especially in the presence of extracellular isoproterenol oxidative stress toxins). Thus, in general, with the isolation of mitochondria from treated animals (or from disease-model animals), much care must be taken [23,54,55]. In these cases, the presence of oxidative stress toxins in the blood and/or extra physiological intramitochondrial calcium is very possible (the role of this extra calcium is to activate the production of mitochondrial ATP, with the purpose of supplying extra energy to all of the processes activated for cellular repair), which (as already discussed) are a source for obtaining isolated mitochondria with bad MEFS of an artefactual type (in particular, this is possibly the origin of the divergent and conflicting scientific results obtained in all the diabetes mitochondrial energetic studies [56,57]). Therefore, we again emphasize: for an adequate isolation of mitochondria from treated or disease model animals, all of what is put forth in the current study and in study [23] is emphatically recommended. Finally, in the literature a greater sensitivity to the activation of the mPT in the isoproterenol-treated heart isolated mitochondria is reported (see for example [40,41]). Much care should be taken with the interpretation of the results of this type of investigation, since in them calcium in vitro is added, but the isoproterenol-treated heart isolated mitochondria already possess, since being in vivo, extra physiological intramitochondrial calcium [30]; so, the isoproterenol-treated heart mitochondria needed much less added calcium for the induction of the mPT [40,41], possibly not because they possess a worse structural-functional state, but because during an isoproterenol treatment, the calcium concentration increases, due to physiological reasons, inside of the heart mitochondria, so the isoproterenol-treated isolated heart mitochondria are more sensitive to exogenous calcium during the induction in vitro of the mPT. It is very important to underline the dilemma in which the mPT studies with isoproterenol-treated heart isolated mitochondria find themselves. For the mPT studies, the use of a calcium chelator in the homogenization buffer is also fundamental, and only the last washing (usually of two washings) should be with a washing buffer without this chelator, but the isoproterenol-treated heart isolated mitochondria will undergo (because they already possess in vivo extra physiological intramitochondrial calcium) negative alterations in their MEFS because of having been washed with a buffer without any calcium chelator.

## 4. Materials and Methods

### 4.1. Experimental Design

The vastly different experimental conditions used in (−)-isoproterenol infarct-like myocardial studies made it difficult to use all of these different experimental conditions at the same time in a new study. In this regard, in the current study the experimental conditions reported by Chagoya de Sanchez and co-workers were selected [7], because this investigation was the most complete (encompassing histological, biochemical, and physiological aspects) of all of those published on HMRA (using isolated mitochondria) upon isoproterenol treatment. The selected experimental conditions from the study [7] were: (i) rats sacrificed at 3, 6, 24, and 48 h after receiving a subcutaneous injection of (−)-isoproterenol-HCL (Sigma; St. Louis, MO, USA) (67 mg/kg body weight); (ii) the times and angular velocities of the centrifuging (as well as the composition of all buffers) for the preparation of the biological samples; and (iii) the statistical comparison of each of the (−)-isoproterenol-treated groups with only the control group (which did not receive any type of injection). Carrying out this same type of statistical comparison is very important for the conclusions of the present investigation to be stronger. In the study [7], no experiments were carried out with any type of placebo group (so the lack of the use in the present study of any kind of placebo group is in accordance with the Three Rs principle in animal experimentation [58]. In this same order of ideas, in the present study histological experiments were not carried out, since the same model of a (−)-isoproterenol infarct-like myocardial lesion is being used as that used by the researchers [7]).

### 4.2. Preparation of the HHs

Female Wistar rats, weighing 200 g, were sacrificed by cervical dislocation, and their hearts were rapidly excised, separated into two or three large sections, placed in cold sucrose (0.29 M) for two to three minutes, squeezed with a manual press (0.8 mm hole-diameter), and gently homogenized with 12 strokes using a Potter homogenizer (Glas-Col; Terre Haute, IN, USA) in a 3.5 mL homogenization buffer/1 g heart (this is the HH), which was conserved at 2–4 °C for the studies of the day). The homogenization buffer (HB) was 180 mM KCL, 10 mM EDTA, and 0.5% BSA, pH 7.2, at 2–4 °C. HHs from (−)-isoproterenol-treated rats were prepared using rats sacrificed 3, 6, 24, and 48 h after receiving a subcutaneous injection of (−)-isoproterenol-HCL (Sigma; St. Louis, MO, USA) (67 mg/kg body weight; in 0.3 mL of 0.9% NaCL). The control group did not receive any type of injection. For the appropriate handling of the laboratory animals, all of the recommendations of the Guide for the Care and Use of Laboratory Animals (1996; National Academy Press) were followed. During the time in which this research was carried out, no committee on the ethics of the handling of laboratory animals existed, so no corresponding institutional approval is available.

### 4.3. Preparation of the CHMs and IHMs

Briefly, the HHs were prepared as described above, and then were complemented with 5 mL of the HB, and finally were centrifuged at 1500× *g* for 10 min. The resulting pellet of this first centrifuging (the first pellet) was discarded and the supernatant was spun at 8500× *g* for 10 min. After this second centrifuging, the supernatant was discarded and the pellet (the second pellet) was suspended in 1 mL of the washing buffer (WB) 180 mM KCL, 0.5% BSA, pH 7.2, at 2–4 °C (these unwashed isolated mitochondria are the CHMs, which were conserved at 2–4 °C for the studies of that day). IHMs were also prepared, which were obtained by first preparing the second pellet (as described above), then suspending this pellet in 8.5 mL of the WB, and finally centrifuging it at 8500× *g* for 10 min. The resulting supernatant was discarded and the pellet (the third pellet) was suspended in 1 mL of the WB (these once-washed isolated mitochondria are the IHMs, which were conserved at 2–4 °C for the studies of that day). The CHMs and IHMs from (−)-isoproterenol-treated rats were prepared using rats sacrificed 3, 6, 24, and 48 h after receiving a subcutaneous injection of (−)-isoproterenol-HCL (Sigma; St. Louis, MO; USA) (67 mg/kg body weight, in 0.3 mL of 0.9% NaCL). The control group did not receive any type of injection.

### 4.4. Determination of the HMRA

The HMRA was determined polarographically with a Clark-type oxygen electrode in the respiratory buffer (RB) 250 mM sucrose, 0.5 mM EGTA, and 3 mM phosphate buffer, pH 7.4. Incubations were carried out in thermostated closed vessels with magnetic stirring. Oxygen rates were measured at 30 °C and obtained in nmol oxygen atoms/mg of protein per minute. The state 2 was the oxygen (O = (1/2)O_2_) consumption per mg of protein per minute (nmol O/minxmg of protein) by HHs, CHMs, or IHMs after glutamate-malate addition (4 mM glutamate plus 1 mM malate). The state 3 was the oxygen (O = (1/2)O_2_) consumption per mg of protein per minute (nmol O/minxmg of protein) after ADP addition (250 μM) into HHs, CHMs, or IHMs, which were at state 2. The RC was calculated as the ratio between state 3 and state 2 (RC = state 3/state 2, dimensionless). The ADP:O was calculated as the ratio of nanomoles of ADP added divided by the nanomoles of oxygen (O = (1/2)O_2_) utilized during state 3 (ADP:O = ADP/O, nmol ADP/nmol O = dimensionless). The MASA was calculated from the product of state 3 and the ADP:O ratio (MASA = state 3 × ADP:O, (nmol O/minxmg of protein) × (nmol ADP/nmol O) = nmol ADP/min × mg of protein). The protein was determined by means of the biuret method [59], using BSA as the standard. All reagents were obtained from Sigma. Here it is worth mentioning the following: it is evident that there are impurities (fragments of other suborganelles, non-mitochondrial ATPases, etc.) in all of the samples of the current study; of all these, the non-mitochondrial ATPases are those that would really affect the records of oxygen consumption. In order to avoid the non-mitochondrial ATPases interference, the oxygen consumption should be determined before adding ADP (after oxidative substrate addition; state 2). Before the addition of ADP, there is only endogenous ATP and its concentration is so low that the activity of the non-mitochondrial ATPases is really very low (or practically zero).

### 4.5. Statistics

All values were expressed as means ± SEM. In the studies with HHs and CHMs, the control group was compared with each of the (−)-isoproterenol-treated groups using the one-way ANOVA method (with the Dunnett’s post test). In the studies with IHMs, the control group and the 3 h (−)-isoproterenol group were compared using the Student’s *t*-test. Differences were considered statistically significant at *p* < 0.05. Statistical analysis was performed using GraphPad PRISM version 2.0 software (San Diego, CA, USA). All the graphics were first made with the help of the GraphPad PRISM version 2.0 software (San Diego, CA, USA) and then these were vectorized using the Adobe Photoshop CS 1990–2003 program (Ann Arbor, MI, USA).

## Data Availability

The datasets generated during and/or analyzed during the current study are not publicly available, because there are a large number of records taken with a very weak line (and therefore it is necessary to be very careful in their scanning). The above-mentioned records are available from the corresponding author on reasonable request.
